# Taming autoimmune thyroiditis: cellular immunomodulation through MSCs, Tregs, and tolDCs

**DOI:** 10.3389/fimmu.2026.1698027

**Published:** 2026-02-18

**Authors:** Ting Peng, Jiangang Wang, Yanhui Lin

**Affiliations:** 1 Health Management Center, The Third Xiangya Hospital, Central South University, Changsha, Hunan, China; 2 Hunan Engineering Technology Research Center for Sub-health Diagnosis and Intervention, Changsha, Hunan, China; 3 Hunan Clinical Medical Research Center for Chronic Disease Health Management, Changsha, Hunan, China

**Keywords:** autoimmune thyroiditis, cellular immunomodulation, mesenchymal stem cells (MSCs), regulatory T cells (Tregs), tolerogenic dendritic cells (tolDCs)

## Abstract

Autoimmune thyroiditis (AIT), typified by Hashimoto’s thyroiditis, represents a prototypical organ-specific autoimmune disorder marked by lymphocytic infiltration, autoantibody production, and progressive thyroid dysfunction. Conventional hormone replacement alleviates hypothyroidism but fails to correct the underlying immune dysregulation. Preclinical models of experimental autoimmune thyroiditis (EAT) consistently demonstrate that these cell-based approaches mitigate inflammatory responses, correct Th17/Treg imbalance, and prevent follicular destruction. Moreover, emerging data on extracellular vesicle–mediated mechanisms and antigen-specific dendritic targeting further underscore the potential for durable immunological reprogramming. This review summarizes recent advances in tolerogenic cellular therapies aimed at restoring immune homeostasis in AIT. Mesenchymal stem cells (MSCs), regulatory T cells (Tregs), and tolerogenic dendritic cells (tolDCs) exert multifaceted immunomodulatory effects via cytokine secretion, metabolic reprogramming, and induction of antigen-specific tolerance, offering a promising immunotherapeutic strategy to modify AIT progression, moving beyond symptomatic relief toward long-term immune tolerance.

## 1 Introduction

Hashimoto’s thyroiditis (HT), a prevalent form of autoimmune thyroiditis (AIT), is a chronic organ-specific disorder marked by progressive lymphocytic infiltration and the generation of thyroid-targeted autoantibodies, ultimately impairing endocrine function of the thyroid gland (1, 2). Microscopically, AIT is characterized by widespread infiltration of immune cell populations, leading to the destruction of thyroid follicular architecture. This replacement of functional parenchyma with lymphoid and stromal elements results in glandular hypertrophy, fibrotic remodeling, and diminished thyroid hormone synthesis, often manifesting as clinical hypothyroidism (3, 4). Despite decades of investigation, the precise immunopathological mechanisms underlying AIT remain only partially elucidated (5). However, extensive evidence supports a multifactorial etiology, in which specific genetic variants play foundational roles. Environmental exposures, aberrant antigen processing, and disrupted cytokine-mediated signaling have also been implicated in modulating the immune cascade involved in disease pathogenesis (6, 7). In addition, the frequent familial aggregation of autoimmune thyroid disorders reinforces the notion of heritable susceptibility, suggesting that both shared genetic determinants and environmental factors contribute to disease development and progression (8).

Current therapeutic strategies for autoimmune thyroiditis primarily aim to alleviate clinical symptoms, with lifelong levothyroxine administration remaining the cornerstone to preserve euthyroid homeostasis. However, disease-modifying immunotherapies have yet to be realized—an issue paralleled in other autoimmune disorders, including rheumatoid arthritis (RA), multiple sclerosis (MS), and systemic lupus erythematosus (SLE) (9–12). In RA, although pharmacological intervention can induce durable remission in approximately one-third of patients, disease recurrence occurs in nearly half upon treatment discontinuation (13, 14). Historically, corticosteroids and cytotoxic compounds have dominated the therapeutic landscape; nonetheless, their broad immunosuppressive profiles heighten vulnerability to opportunistic infections and oncogenic transformation (14). This review focuses on delineating the immunoregulatory functions and therapeutic prospects of tolerogenic cellular therapies in the context of AIT.

## 2 Immunopathogenic mechanisms underlying autoimmune thyroiditis

AIT arises from a breakdown of self-tolerance to thyroid-specific antigens, triggering chronic inflammation and glandular dysfunction (15, 16). A central immunopathological hallmark is the skewed Th17/Treg axis. Th17 cells, via IL-17, IL-22, and GM-CSF, orchestrate neutrophil recruitment and propagate tissue-destructive inflammation (17, 18). In contrast, FoxP3^+^ regulatory T cells (Tregs) exert immunosuppressive effects through IL-10 and TGF-β (19, 20). A persistent Th17/Treg imbalance is consistently observed in both Hashimoto’s thyroiditis (HT) patients and experimental autoimmune thyroiditis (EAT) models, correlating with disease severity (21, 22). Mechanistic insights have revealed epigenetic and metabolic perturbations underlying impaired Treg function. Notably, the NAD^+^-dependent histone deacetylase SIRT1 destabilizes FoxP3 by promoting its deacetylation, compromising Treg suppressive capacity (23–25). Elevated SIRT1 levels in CD4^+^ T cells from HT patients associate with reduced FoxP3 expression and attenuated regulatory activity (25). Pharmacological inhibition of SIRT1 restores FoxP3 acetylation and expression, enhances Treg differentiation, and reinstates immune suppression—underscoring its critical role in AIT pathogenesis (26).

In parallel, dendritic cell (DC) dysfunction contributes to immune dysregulation. Activated conventional DCs upregulate costimulatory molecules and pro-inflammatory cytokines, driving autoreactive T cell expansion (27, 28). In contrast, tolerogenic DCs (tolDCs)—characterized by IL-10 secretion, IDO activity, and expression of immune checkpoints (ILT3, ILT4)—promote antigen-specific Treg induction and dampen effector responses (29–32). The functional insufficiency of tolDCs further tips the immune balance toward autoimmunity (33, 34). These intersecting mechanisms—Th17/Treg disequilibrium, SIRT1-mediated FoxP3 suppression, and failed DC tolerance—form the immunological framework of AIT.

## 3 Tolerogenic cell populations

### 3.1 Mesenchymal stem cells

MSCs are multipotent stromal cells capable of differentiating into osteogenic, adipogenic, and chondrogenic lineages, and are identified by expression of CD105, CD73, and CD90, but not hematopoietic markers such as CD45, CD34, or CD14 (35–37). Beyond their regenerative properties, MSCs exert potent immunomodulatory effects through paracrine secretion and direct cell–cell interactions, positioning them as promising candidates for treating autoimmune diseases (38, 39). They are readily isolated from diverse sources, including adipose tissue, amniotic membrane, and umbilical cord matrix (36). MSCs mediate immune suppression by releasing soluble factors such as indoleamine 2,3-dioxygenase (IDO), prostaglandin E_2_ (PGE_2_), and transforming growth factor-β (TGF-β), which collectively suppress effector T cell proliferation and promote Treg expansion (40). In AIT, MSCs restore Th17/Treg balance by activating the IDO–kynurenine–aryl hydrocarbon receptor (AhR) axis and the PGE_2_–cAMP–TGF-β–FoxP3 pathway. IDO catabolizes tryptophan into kynurenine, triggering GCN2–eIF2α signaling that inhibits effector T cell expansion, while kynurenine engages AhR to upregulate FoxP3 and inhibit RORγt-driven Th17 differentiation (41, 42). Concurrently, PGE_2_ binds EP2/EP4 receptors on T cells, suppressing IL-2 production via cAMP–PKA signaling, and in the presence of TGF-β, promotes the induction and stabilization of FoxP3^+^ Tregs (43). In addition to T cell modulation, MSCs reprogram innate immune cells. They condition dendritic cells (DCs) toward a tolerogenic phenotype characterized by low CD80/CD86 and MHC II expression, reduced IL-12p70, and elevated IL-10 production—mediated through PGE_2_, TGF-β, TSG-6, and nitric oxide (44, 45). MSCs also promote M2 macrophage polarization (high CD206, IL-10), reinforcing an anti-inflammatory environment in the thyroid (46, 47). Furthermore, MSCs inhibit B cell proliferation, impair plasma cell differentiation, and reduce proinflammatory signaling from CD4^+^ T cells, collectively dampening autoantibody production and limiting tissue damage (48, 49). Together, these findings underscore MSCs’ multifaceted capacity to recalibrate both innate and adaptive immunity. By simultaneously modulating T cells, DCs, macrophages, and B cells, MSCs offer a comprehensive immunotherapeutic strategy for restoring peripheral tolerance and counteracting pathogenic autoimmunity in AIT.

### 3.2 Regulatory T cells

Tregs, defined by CD4, CD25, and FoxP3 co-expression, are essential for immune self-tolerance. They suppress effector T cell activation and pro-inflammatory cytokine secretion, and their depletion or FoxP3 dysfunction is causally linked to autoimmune and allergic diseases (50, 51). Through multiple suppressive mechanisms—including inhibition of IL-2 secretion and T cell proliferation—Tregs modulate both innate and adaptive immune compartments, affecting T cells, B cells, dendritic cells, macrophages, and mast cells (52–54). In AIT, Tregs are numerically and functionally deficient (55–57), comprising only 5–10% of CD4^+^ T cells and exerting suppression via cytokines such as IL-10, TGF-β, and IL-35. These mediators inhibit antigen-driven cytokine cascades and immune effector cell activation (58). Clinically, patients with AIT display reduced Treg frequencies in both thyroid tissues and peripheral blood compared to healthy individuals (59), and murine EAT models demonstrate that Treg depletion worsens disease, whereas adoptive transfer alleviates pathology (60).

FoxP3 functions as the lineage-defining transcription factor essential for regulatory T cell (Treg) stability and immunosuppressive function (61, 62). Its expression is tightly governed by TGF-β, which promotes Treg differentiation while concurrently inhibiting pro-inflammatory Th cell fates. In autoimmune thyroiditis (AIT), this regulatory axis is disrupted: reduced TGF-β and increased IL-12 (via its p40 subunit) synergistically suppress FoxP3 transcription while promoting nitric oxide production, thereby undermining Treg identity (25, 63). Murine studies consistently underscore the indispensability of FoxP3 for preserving immune tolerance (64). Beyond transcriptional regulation, post-translational modification of FoxP3—particularly via SIRT1, a class III NAD^+^-dependent histone deacetylase—has emerged as a pivotal mechanism of Treg dysfunction in AIT. In Hashimoto’s thyroiditis (HT), CD4^+^ T cells exhibit elevated SIRT1 expression, which inversely correlates with FoxP3 levels and Treg-mediated suppression (65–67). SIRT1 deacetylates FoxP3, compromising its stability and transcriptional activity. Strikingly, pharmacological inhibition of SIRT1 with Ex-527 restores FoxP3 acetylation, enhancing both mRNA and protein expression. This intervention not only expands the functional Treg compartment but also reinstates immunological balance (25). Therapeutic strategies targeting the SIRT1–FoxP3 regulatory axis thus represent a compelling approach to restoring Treg homeostasis. Preclinical evidence supports the notion that re-establishing FoxP3 function mitigates disease severity in AIT, providing a rational framework for future translational applications of cell-based immunotherapies (68).

### 3.3 Tolerogenic dendritic cells

**Figure 1:**
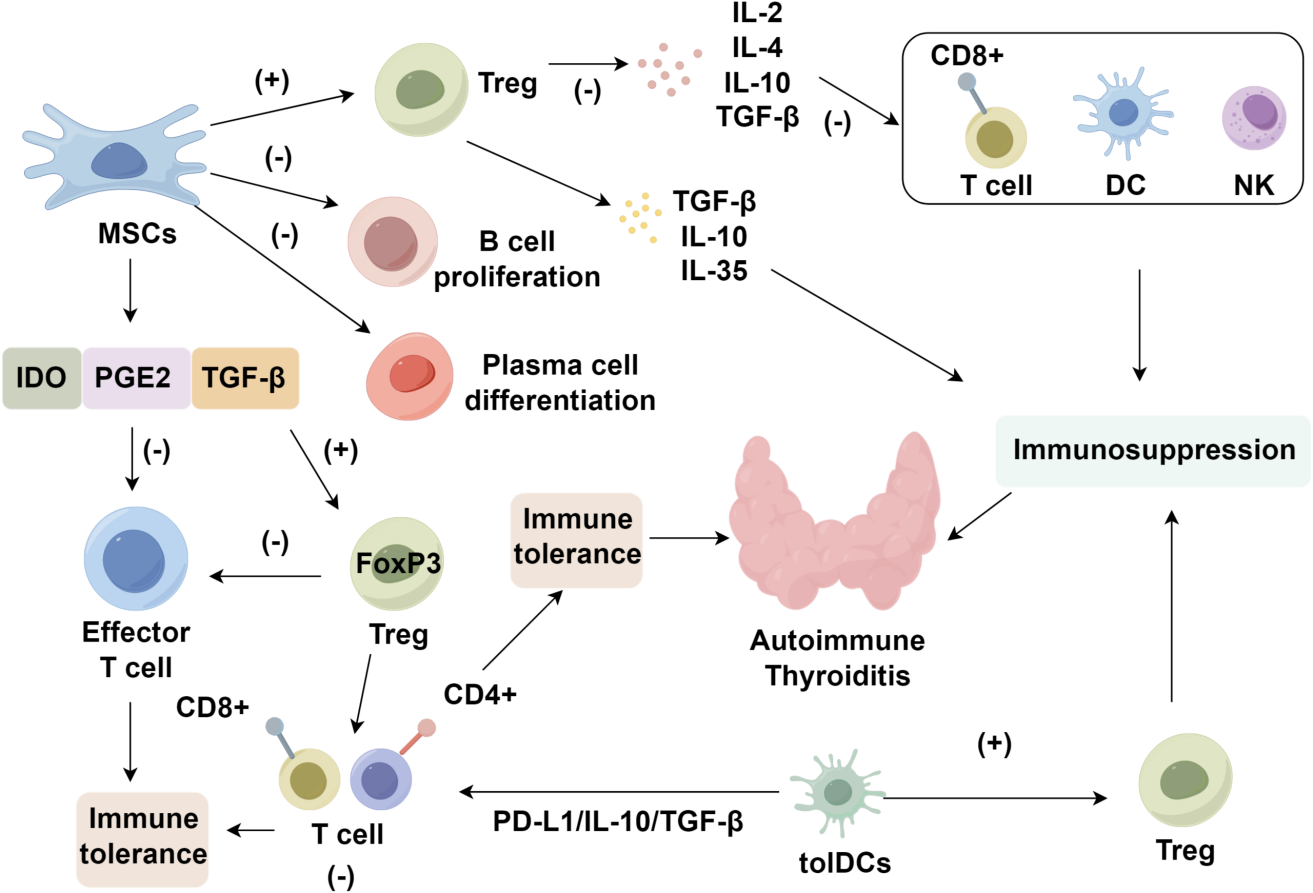
Roles of tolerogenic cell in autoimmune thyroiditis. This figure illustrates the immunomodulatory pathways by which mesenchymal stem cells (MSCs), regulatory T cells (Tregs), and tolerogenic dendritic cells (tolDCs) suppress autoreactive immune responses in autoimmune thyroiditis. MSC-derived factors (IDO, PGE_2_, TGF-β) inhibit effector T cells, B cell proliferation, and plasma cell differentiation while promoting Treg expansion. Tregs secrete IL-10, TGF-β, and IL-35 to dampen CD8^+^ T cells, dendritic cells (DCs), and NK cells. tolDCs further induce Tregs via PD-L1 and IL-10 signaling. These synergistic pathways collectively re-establish immune tolerance and mitigate thyroid autoimmunity.

DCs, originating from hematopoietic precursors, are central antigen-presenting cells (APCs) that initiate adaptive immunity through migration to secondary lymphoid tissues and engagement with lymphocytes via upregulated costimulatory and adhesion molecules (69–71). In autoimmune settings, DCs often adopt a pro-inflammatory phenotype, secreting elevated cytokines and activating autoreactive effector T cells, thereby intensifying tissue damage. Their functional polarity—toward either immunogenic or tolerogenic states—is governed by antigen exposure, maturation signals, and cytokine context (72). While conventional DCs encompass classical and plasmacytoid subsets (73), tolDCs constitute an induced, plastic state essential for central and peripheral immune tolerance. *In vivo* targeting of DEC-205–tagged self-antigens to DCs induces transient T cell activation followed by deletion or anergy (74–76). tolDCs are marked by diminished CD80/CD86, elevated ILT3/ILT4, and IDO expression, driving Treg induction and effector T cell suppression (77, 78). This phenotype is maintained via STAT3-mediated IL-10 transcription and AHR signaling, reinforcing tolerogenic gene networks (79, 80). Ex vivo generation of tolDCs involves culturing monocytes or CD34^+^ precursors with GM-CSF and IL-4, followed by conditioning with immunomodulators, including IL-10, dexamethasone, vitamin D3, or rapamycin, to induce a regulatory profile (81–83). These cells exhibit low MHC-II and costimulatory marker expression, alongside increased PD-L1, ILT3, and IL-10. However, clinical translation faces key hurdles (84). Immunoregulatory agents including IL-27, retinoic acid derivatives, and AHR ligands have been shown to potentiate tolDC differentiation and antigen-specific hyporesponsiveness (85–88). In AIT, the breakdown of tolerance to thyroid autoantigens (TPO, Tg, TSH-R) leads to autoreactive T cell expansion, autoantibody production, and IFN-γ/TNF-α–driven inflammation (89, 90). tolDCs offer a targeted approach to reestablish tolerance by presenting thyroid antigens in a non-inflammatory context, inducing IL-10–secreting CD4^+^CD25^+^ Tregs, and stabilizing this phenotype via STAT3 and AHR pathways. Thus, tolDC-based therapy may interrupt the self-amplifying immune cascade in AIT, providing a rational, cell-based strategy for immune re-education (**Figure 1**).

## 4 Tolerogenic cell therapy in autoimmune thyroiditis

Although the precise *in vivo* mechanisms by which exogenously delivered tolerogenic cell populations confer therapeutic benefits remain incompletely defined, accumulating evidence highlights their central immunomodulatory roles (84, 91). Among them, mesenchymal stem cells (MSCs), regulatory T cells (Tregs), and tolerogenic dendritic cells (tolDCs) exhibit a shared capacity for directed migration—from the site of administration to secondary lymphoid organs and subsequently to sites of inflammation or tissue damage—where they initiate context-specific regulatory and reparative responses (92–94). Tregs, for instance, constitutively express L-selectin, enabling entry into peripheral lymphoid structures. Upon antigen encounter, activated Tregs significantly upregulate chemokine receptors (CCR2, CCR4, CCR5, CCR8) and adhesion molecules (CD54, LFA-1), enhancing their capacity to traffic into inflamed microenvironments and exert local immunosuppressive effects (95–97). Analogously, MSCs and tolDCs exhibit comparable migratory behaviors, reinforcing their capacity for targeted immune modulation (70, 98, 99). This directed homing to inflammatory loci appears to be a prerequisite for the efficacy of tolerogenic cell-based therapies, as it enables localized immunoregulation through intrinsic cellular mechanisms (70, 100, 101).

### 4.1 Mesenchymal stem cell–mediated modulation in EAT

To model autoimmune thyroiditis (AIT), CBA/J mice were immunized with porcine thyroglobulin in Freund’s adjuvant, inducing robust autoreactive T and B cell responses and subsequent thyroidal destruction (102). Administration of human amniotic membrane–derived mesenchymal stem cells (HA-MSCs) significantly reduced serum levels of anti-thyroperoxidase (TPOAb) and anti-thyroglobulin (TgAb), irrespective of dosing regimen. This therapeutic effect was accompanied by expansion of IL-10–producing B10 cells and a corresponding reduction in pro-inflammatory Th17 subsets, implicating MSCs in modulation of the B10–Th17 axis to suppress autoimmunity (102, 103). Concordant results were observed in a rat model of experimental autoimmune thyroiditis (EAT) treated with human umbilical cord–derived MSCs, which exhibited reduced lymphocytic infiltration and preserved follicular architecture, along with Th17 downregulation (104). Similarly, adipose-derived MSCs (ADSCs) in C57BL/6 EAT mice suppressed systemic pro-inflammatory cytokines (IFN-γ, IL-6, IL-2, and TNF-α) and decreased the IFN-γ/IL-4 ratio (105, 106). ADSC treatment also led to diminished CD11b^+^ splenocytes and CD4^+^IL-17^+^ T cells, alongside an increase in splenic Treg populations and reduction in circulating autoantibodies (107, 108). Beyond cellular therapy, MSC-derived extracellular vesicles (EVs) recapitulate many immunoregulatory effects of parental cells. These vesicles deliver bioactive microRNAs, immunomodulatory proteins, and lipid mediators that collectively restrain autoreactive T cell activity, inhibit pro-inflammatory cytokine production, and facilitate Treg expansion (104, 109–111). Additionally, MSCs secrete trophic factors such as VEGF, HGF, and FGF-2, which support neovascularization, extracellular matrix remodeling, and thyroid follicular regeneration (112, 113). Although clinical translation remains nascent, these converging preclinical findings underscore the potent immunotolerogenic capacity of MSC-based strategies in mitigating pathogenic lymphocytic responses in AIT models (109, 114, 115).

### 4.2 Regulatory T cells: immune modulation within EAT

The immunosuppressive function of regulatory T cells (Tregs) was first rigorously delineated by Hori, who identified them as a distinct subset of CD4^+^ T lymphocytes characterized by high CD25 expression and the lineage-defining transcription factor FoxP3 (116). Initially termed natural Tregs (nTregs), these cells exhibit potent inhibitory effects on effector T-cell proliferation, activation, and pro-inflammatory cytokine production. In a CBA/J transgenic model of autoimmune thyroiditis, Verginis demonstrated that stimulation of CD4^+^CD25^+^ Tregs markedly suppressed thyroglobulin-induced disease development (31). Subsequent studies across various preclinical models have consistently validated the indispensable role of Tregs in restraining thyroid-specific autoimmunity (117–119). Clinically, Treg frequencies in untreated individuals mirrored those of healthy controls but were significantly diminished relative to patients receiving long-term therapy (120), suggesting treatment-associated Treg expansion. Notably, Tregs can diversify into phenotypically and functionally distinct subsets, such as CD4^+^CD69^+^ and CD4^+^NKG2D^+^ cells, which suppress immune responses through mechanisms involving IL-10, glucocorticoid-induced TNF receptor (GITR), and TGF-β (121, 122). In experimental autoimmune thyroiditis, the therapeutic efficacy of Tregs has been attributed to their capacity to reprogram intracellular signaling networks regulating the Th17/Treg axis (123), thereby attenuating pro-inflammatory cytokine production and restoring immune homeostasis. Critically, the Th17/Treg balance has emerged as a key immunological determinant in the pathogenesis of autoimmune thyroid diseases. While Th17 cells drive inflammation and tissue injury, Tregs mitigate these effects by limiting effector T-cell activation and expansion (124). In Graves’ disease, elevated Th17 cell frequencies and increased IL-17 levels correlate with the severity of ophthalmopathy (125, 126). Similarly, Marazuela identified augmented Th17 cell differentiation and IL-17 output in autoimmune thyroiditis (127). Taken together, these findings underscore the therapeutic promise of strategies aimed at augmenting Treg abundance—whether via adoptive transfer or pharmacological modulation—as a cornerstone of tolerogenic cellular immunotherapy for thyroid autoimmunity.

### 4.3 Tolerogenic cell-based interventions in autoimmune thyroiditis

In a seminal study, Verginis et al. (31) demonstrated that bone marrow–derived DCs conditioned with TNF-α and Tg acquired a semi-mature, tolerogenic phenotype capable of suppressing EAT in mice. These tolDCs selectively expanded Tg-specific CD4^+^CD25^+^ Tregs *in vivo*, characterized by increased expression of Treg-associated markers and IL-10 secretion upon antigen encounter, highlighting their role in antigen-specific immune tolerance. Supporting these findings, Vasu et al. (128) showed that pretreating DCs with granulocyte–macrophage colony-stimulating factor (GM-CSF) prior to immunization with murine Tg conferred a regulatory phenotype that mitigated thyroiditis, in contrast to the exacerbated pathology observed with Flt3 ligand (Flt3-L)–conditioned DCs. GM-CSF–modulated DCs promoted the expansion of IL-4^+^ and IL-10^+^ lymphocytes upon re-exposure to Tg and increased CD4^+^CD25^+^ T cell frequencies *in vivo*, signifying a shift toward a tolerogenic immune landscape suppressing effector responses. Together, these studies underscore the capacity of tolDCs to restore tolerance in AIT by eliciting IL-10–secreting Tregs. In parallel, recent preclinical efforts have emphasized the need for optimized protocols that can reliably generate tolDCs with consistent immunoregulatory function across different donor sources (78, 129, 130). Studies employing IL-10/dexamethasone–conditioned human monocyte-derived DCs have demonstrated durable suppression of autoreactive T cell responses *in vitro*, yet the long-term efficacy and tolerogenic stability of these cells in inflammatory autoimmune environments remain under investigation (32, 131). Emerging manufacturing platforms now explore semi-automated culture systems, closed-loop GMP-compliant bioreactors, and synthetic biomaterials to support tolDC scalability and phenotype retention during expansion and storage (132, 133). These innovations will be essential to bridge the gap between bench and bedside in AIT-directed tolDC therapy. Expanding on DC-based strategies, Coppola et al. (134) explored the immunomodulatory potential of fibroblast-like limbal stem cells (f-LSCs) in AIT. *In vitro* co-culture of patient-derived T cells with autologous f-LSCs—harboring low immunogenicity and enriched for immunosuppressive mediators including PD-L1, PD-L2, IDO, IL-6, and MCP-1—resulted in attenuation of monocyte activation and sustained CD69 expression, suggesting robust and durable immune suppression. Notably, f-LSCs lacked MHC class II and costimulatory molecules, further supporting their tolerogenic profile. Collectively, these findings point to promising antigen-specific and cell-based immunotherapies for AIT.

## 5 Conclusion

Autoimmune thyroiditis (AIT) is a chronic immune-mediated disorder for which current treatments remain palliative, offering only symptomatic relief without addressing the underlying immunopathogenic mechanisms. Recent advances in tolerogenic cell-based therapies, including mesenchymal stromal cells (MSCs), regulatory T cells (Tregs), and tolerogenic dendritic cells (tolDCs), have demonstrated the capacity to recalibrate aberrant immune responses through distinct yet potentially complementary mechanisms. Specifically, MSCs exert anti-inflammatory effects via the secretion of immunosuppressive cytokines and enzymes; Tregs enforce peripheral tolerance by suppressing effector T cell activity; and tolDCs promote antigen-specific Treg induction and expansion.

Although combinatorial strategies may theoretically enhance therapeutic efficacy, the existence of true synergy remains speculative and awaits systematic validation in preclinical models. Strategic clinical translation hinges on addressing several key bottlenecks. MSCs currently exhibit the most mature safety and manufacturing profiles, while tolDCs confer antigen specificity but are hampered by phenotypic instability and limited *in vivo* persistence. Treg-based therapies, although potent, face hurdles in scalable expansion and sustained phenotype maintenance. Progress is further constrained by the absence of standardized animal models that faithfully recapitulate human immunopathology, limited availability of *in vivo* cell-tracking platforms, and the lack of direct comparative analyses between single-cell and combinatorial approaches. Moreover, the long-term safety profile and immunogenicity of repeated cell-based administrations remain incompletely understood. Advancing these approaches toward clinical applicability will require a staged roadmap encompassing mechanistic optimization, dose titration, immune monitoring, and rigorously designed early-phase trials. Collectively, tolerogenic cell therapies offer a promising avenue to restore immune equilibrium in AIT and hold the potential to shift the therapeutic paradigm from symptom management toward durable immunomodulation.
